# Human amniotic mesenchymal stromal cells promote bone regeneration via activating endogenous regeneration

**DOI:** 10.7150/thno.45249

**Published:** 2020-05-15

**Authors:** Fei Jiang, Wenjie Zhang, Mingliang Zhou, Zhixuan Zhou, Ming Shen, Ning Chen, Xinquan Jiang

**Affiliations:** 1 Jiangsu Key Laboratory of Oral Diseases, Nanjing Medical University, No. 140, Han Zhong Road, Nanjing 210029, China; 2 Department of General Dentistry, Affiliated Hospital of Stomatology, Nanjing Medical University, No. 136, Han Zhong Road, Nanjing 210029, China; 3 Department of Prosthodontics; Shanghai Engineering Research Center of Advanced Dental Technology and Materials; Shanghai Key Laboratory of Stomatology & Shanghai Research Institute of Stomatology; National Clinical Research Center for Oral Diseases; Shanghai Ninth People's Hospital, College of Stomatology, Shanghai Jiao Tong University School of Medicine, No. 639 Zhizaoju Road, Shanghai 200011, China

**Keywords:** HAMSCs, paracrine function, M2 macrophage polarization, endogenous regeneration, vascularized bone regeneration

## Abstract

**Rationale**: The effectiveness of stem cell based-therapy for bone regeneration has been demonstrated; yet, clinical application of autologous stem cells is still limited by invasive acquisition, long culture processes, and high cost. Besides, it remains controversial whether autologous stem cells could directly participate in tissue repair after differentiation. Thus, increasing allogeneic stem cells have been developed into drugs to indirectly activate endogenous regeneration and induce tissue regeneration. Human amniotic mesenchymal stromal cells (HAMSCs) have been extensively studied, showing multiple regulatory functions, but mechanisms of HAMSCs in promoting bone regeneration are remain unclear.

**Methods**: Proteome profile of HAMSCs and their functions on vascularized bone regeneration were investigated *in vitro*, while rabbit cranial defect model was used to further detect the effects of bone formation *in vivo*.

**Results**: HAMSCs secrete many osteogenic, angiogenic, and immunomodulatory cytokines. *In vitro*, HAMSCs can promote human bone-marrow mesenchymal stromal cells (HBMSCs) migration and osteogenic differentiation; promote the capillary-tube formation of human umbilical vascular endothelial cells (HUVECs), induce HUVECs migration and pro-angiogenic genes expression, and promote M2 macrophage polarization. Further, *in vivo* studies suggested that transplanted HAMSCs could survive and induce M2 macrophages to secrete bone morphogenetic protein-2 (BMP-2) and vascular endothelial growth factor (VEGF) in rabbits' skull defects at an early stage, and, in turn, promote more new bone formation.

**Conclusion**: HAMSCs have good biocompatibility and paracrine function to promote bone repair by stimulating endogenous regeneration.

## Introduction

Stem cell-based tissue regeneration has been extensively studied over the last two decades, showing certain curative effects 1, 2. Still, *in vitro* methods for collection, isolation, and maintenance of these cells are challenging, which may increase patients' pain and costs 3. Moreover, it is still controversial whether stem cells could participate in healing processes directly. Along with endogenous regeneration proposed and its application expanded, an increasing number of researchers began to focus on how to improve the regeneration effect by activating and regulating endogenous factors 4, 5. Some studies have shown that allogeneic stem cells could have an essential role in inflammation and immune regulation to promote tissue regeneration or treat diseases 6, 7. Thus, it provides a new strategy for regenerative medicine by using allogeneic stem cells to regulate the local microenvironment and activate endogenous regeneration.

Human amniotic mesenchymal stromal cells (HAMSCs) are isolated from amniotic membrane (AM) of the human term placenta that is usually discarded after childbirth. HAMSCs can be harvested in a noninvasive way and without ethical controversy 8. Since human term placenta which plays a key role in maintaining maternal-neonatal tolerance, HAMSCs from this tissue have superior immunomodulatory properties 9. In addition, previous studies found that HAMSCs are potentially implicated in tissue regeneration by identifying factors produced by them, including immunomodulatory factors important for the resolution of inflammation (MCP-1, IL-6, IL-8), growth and angiogenic factors important for tissue remodeling (Angiogenin, CXCL1, VEGF, PDGF) 8. Nevertheless, the curative effect and regulatory mechanism of HAMSCs on bone regeneration still remain unknown.

In this study, we investigated the treatment effect of HAMSCs and its underlying mechanism on promoting bone regeneration *in vivo* and *in vitro*. The new mechanisms of HAMSCs in tissue regeneration could be a powerful impetus for their mass production as a drug and their commercial application.

## Methods

### Proteome Profile of HAMSCs and HBMSCs

Human XL Cytokine Array Kit (ARY022) was used to detect the cytokines secreted by HAMSCs in their culture media following the manufacturer's instructions. The Kit was purchased from R&D System (USA). We analyzed the differences between HAMSCs and HBMSCs, and further categorized the cytokines secreted by HAMSCs according to their roles in osteogenesis, angiogenesis and immune regulation.

### Osteoinduction and Chemotaxis of HAMSCs for HBMSCs

#### HBMSCs osteo-differentiation assay

To determine whether HAMSCs could enhance osteo-differentiation of HBMSCs, we inoculated HBMSCs into 24-well plates. We applied the osteogenic induction medium to induce HBMSCs, and introduced HAMSCs by transwell to observe the synergistic effect of HAMSCs on promoting osteogenic differentiation of HBMSCs.

#### HBMSCs migration assay

HBMSCs migration assay was performed by 24-well plates with 8 μm transwell upper chambers. Briefly, HAMSCs were seeded in the lower chambers of 24-well the co-culture plates. When the confluence of HAMSCs in the lower chamber reached 90%, HBMSCs were seeded in the upper chambers at a density of 1 × 10^4^ cells/well; these cells were co-cultured in serum-free medium 10. After 24 h and 48h incubation, nonmigrating cells on the upper side of the insert transwell membrane were wiped off using a cotton bud, and the cells on the lower surface of the same membrane were fixed in methanol and stained with trypan blue. The number of migrating cells to the lower surface of the membrane was counted in three random fields under a light microscope 10. The analysis was performed three times.

#### Polymerase chain reaction assay for HBMSCs osteogenic markers

After co-culturing HBMSCs with HAMSCs for 3d and 7d, the HBMSCs RNA was extracted by using TRIzol reagent (Sigma, USA). The RNA was then transcribed into cDNA by the PrimeScript RT master Mix (Takara, Japan). Osteogenesis-related genes, including runt-related transcription factor 2 (Runx2), alkaline phosphatase (ALP), and collagen type I α 1 chain (Col1A1) were assayed using a real-time PCR system (LightCycler® 480Ⅱ; Roche, Switzerland). The relative gene expression levels were determined by the 2^-ΔΔCt^ method. Glyceraldehyde-3-phosphate dehydrogenase (GAPDH) was selected to normalize the expression levels of the target genes. The results were presented as the fold increase relative to that of the control group. All assays were carried out in triplicate. The PCR primer sequences used in this study are listed in Table S1.

### Angiogenesis and Chemotaxis of HAMSCs for HUVECs

#### Capillary-like tube formation assay

The formation of HUVECs into capillary-like structures on Matrigel (B&D system, USA) was evaluated as previously described 9, 16. The medium in which HAMSCs were cultured for over 48h was collected and stored at -80℃ for capillary-like tube formation assay. The media was named HAMSC-conditional media (HAMSC-CM). Before the experiment, 96-well plates were coated with Matrigel and put at 37°C in a humidified atmosphere for at least 30min. Then, trypsin-harvested HUVECs were resuspended in α-MEM, HAMSC-CM and EBM-2 respectively and seeded onto the plated Matrigel (2 × 10^4^ cells per well). The α-MEM culture condition was used as a negative control, while the EBM-2 culture condition was used as a positive control. Images of the formation of capillary-like structures were obtained after 6, 24 h and 60h with a microscope (Leica, Germany) at 100
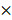
magnification. Tubular structures were quantified by manually counting branch points and length of HUVEC sprouting in randomly selected fields at 100
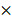
magnification.

#### HUVECs migration assay

HUVECs migration assay was performed by 24-well plates with 8 *μ*m transwell upper chambers (Corning, USA). Briefly, HAMSCs were seeded in the lower chambers of 24-well the co-culture plates. When the confluence of HAMSCs in the lower chamber reached 90%, HUVECs were seeded in the upper chambers at a density of 1 × 10^4^ cells/well, and these cells were co-cultured in serum-free medium 10. After 24 h and 48h incubation, nonmigrating cells on the upper side of the insert transwell membrane were wiped off using a cotton bud, and the cells on the lower surface of the same membrane were fixed in methanol and stained with trypan blue. The number of migrating cells to the lower surface of the membrane was counted in three random fields under a light microscope 10. The analysis was performed three times.

#### Polymerase chain reaction assay for HUVECs angiogenic markers

After HUVECs co-culturing with HAMSCs for 3d and 7d, the HUVECs RNA was extracted by using TRIzol reagent (Sigma, USA). The RNA was then transcribed into cDNA by the PrimeScript RT master Mix (Takara, Japan). Angiogenesis-related genes, including angiopoietin-1 (ANG-1), angiopoietin-2 (ANG-2), vascular endothelial growth factor (VEGF) and fibroblast growth factor 2 (FGF-2) were assayed using a real-time PCR system (LightCycler® 480Ⅱ; Roche, Switzerland). The relative gene expression levels were determined by the 2^-ΔΔCt^ method. Glyceraldehyde-3-phosphate dehydrogenase (GAPDH) was selected to normalize the expression levels of the target genes. The results were presented as the fold increase relative to that of the control group. All assays were carried out in triplicate. The PCR primer sequences used in this study are listed in Table S1.

### Role of HAMSCs on Macrophage Polarization

The macrophage lineage diversification and plasticity are key aspects of their functionality, such as inflammatory M1 to anti-inflammatory or pro-regenerative M2 cells. Microenvironmental signals within the injury modulate the polarization of macrophages. To determine the effect of HAMSCs paracrine function on the polarization of macrophages, we inoculated HAMSCs with macrophages (RAW264.7 cell line) 11 and co-cultured them directly 12 for 3 days. The samples were fixed with 4% paraformaldehyde for 15min and blocked with 5% donkey serum for 30min at room temperature. Then, samples were incubated with anti-iNOS (1:100, ab15323) primary antibody from Abcam (UK) and anti-MMR (1:100, AF2535) primary antibody from R&D system (USA) over night at 4°C. Alexa Fluor^®^ 488-conjugated Donkey anti-rabbit IgG (1:200) and Alexa Fluor^®^ 594-conjugated Donkey anti-goat IgG (1:200) were used as the secondary antibody from Invitrogen (USA). The cell nuclei were stained by DAPI (Sigma, USA) for 5min at room temperature. The iNOS- and MMR-positive cells were investigated by a fluorescent microscope (Olympus Corporation, Japan) with higher magnifications (400×).

### Animal Experiment and Samples Preparation

The study was approved by the Institutional Animal Care and Use Committee of Nanjing Medical University (No. PJ2014-079-001). Six C57BL/6 mice and eighteen New Zealand white rabbits were used for the experiment. The animals were anaesthetized by intravenous injection of pentobarbital sodium (1.5 mg/kg) before surgery procedure.

We fabricated two standardized round bone defects in the right and left cranial bone of one rabbit with a 1cm diameter trephine. Plasminogen-depleted human fibrinogen solution was prepared by serum-free media at a concentration of 5 mg/ml and filtered for sterility. According to the filling materials, all cranial defects were divided into four groups on average (n=3 per group at each time-point): Control group (as negative control), HAMSC group, Bio-Oss group (as positive control), and HAMSC/Bio-Oss group (Figure 5C). The HAMSCs for this animal experiment were trypsinized and resuspended by fibrinogen solution with 0.5×10^6^ HAMSCs per 100 μL. The fibrinogen-HAMSC solution was catalyzed by thrombin solution (50U/mL; Sigma, USA) in a ratio of 20:1 to form the HAMSC-gel. To detect the HAMSC survival *in vivo*, the Dil-HAMSC-gel was subcutaneously injected into mice. After 14d, the samples were collected and prepared by the frozen section. Then, the frozen sections were stained by the human-specific marker to confirm the survival of transplanted HAMSCs. In the bone defects of rabbits' cranial department, the HAMSC-gel was injected before crosslinking for the HAMSC group and HAMSC/Bio-Oss group. The rabbits were sacrificed by euthanasia at 2 weeks (2w), 4 weeks (4w) and 12 weeks (12w) after operations respectively. The skulls were then collected and fixed in 4% phosphate-buffered formalin solution.

All samples in the 2-week group and 4-week group were decalcified, embedded in paraffin and sectioned into 4 mm thick sections. The cranial defect samples in the 12-week group were equally divided into two parts equally by coronal direction. One half of the samples in the 12-week group were also decalcified, embedded in paraffin and cut into 4 μm thick sections; the other half of the samples were embedded in polymethylmethacrylate (PMMA) and cut into 150 mm thick sections using a microtome (Leica, Germany) 13. These sections were gradually ground and polished to thickness of 40 mm for observing sequential fluorescent labeling.

### Micro-CT Measurement

The morphology of the reconstructed cranial samples was assessed using an animal micro-CT scanner (SkyScan 1176 scanner, SkyScan, Kontich, Belgium). Briefly, the specimens were scanned with some parameters, including an X-ray tube potential of 80 kV, a tube current of 0.45 mA, and 18-μm voxel resolution. After micro-CT scan, the reconstructed micro-CT images were analyzed using NRecon v1.6 and CTAn v1.13.8.1 software. Figure 7 showed the representative micro-CT images in 4w-group and 12w-group. Bone volume (BV) and Bone volume/tissue volume (BV/TV) of Control groups and HAMSC groups were evaluated; these data of Bio-Oss groups and HAMSC/Bio-Oss groups were not displayed in micro-CT analysis due to X-ray blocking interference of Bio-Oss particles.

### Immunofluorescence Examination

Immunofluorescence staining of human MHC class I+HLA A+HLA B (1:100, ab134189, Abcam, UK) was performed to determine the survival of transplanted HAMSCs in subcutaneous regions of mice cranium and bone defect regions of rabbit cranium. The expressions of iNOS and MMR in 2w-group rabbit cranial defects were detected by immunofluorescence staining to show local macrophage infiltration in early osteogenic stage. Meanwhile, we further co-stained MMR with VEGF and BMP2 to confirm whether macrophages participated in regulating local precursor cells to enhance bone regeneration. ALP and CD31 were then chosen to show the effects of vascularized bone regeneration in four groups. Briefly, paraffin sections (4 μm) were deparaffinized, washed three times in PBS for 5 min, blocked with 5% serum for 30 min and incubated overnight at 4 °C with all primary antibodies. After rinsing three times with PBS, the slides were incubated with Alexa Fluor^®^ 488 and 594 secondary antibodies (1:200, Invitrogen, USA) respectively. After washing secondary antibody three times in PBS, the cell nuclei were stained by DAPI (Sigma, USA) for 5min at room temperature. The positive stained cells were observed by a fluorescent microscope (Olympus Corporation, Japan) with higher magnifications (400×) in five random fields for each section. The area of positive stained cells was captured and calculated by Image J software for statistical analysis 14. The primary antibodies for iNOS (1:100, ab49999), VEGF (1:100, ab1316) and BMP2 (1:100, ab6285) were purchased from Abcam (UK), while primary antibodies for ALP (1:50, AF2910) and MMR (1:100, AF2535) were purchased from R&D system (USA). The primary antibodies for CD31 (1:50, NB100-64796) were purchased from NOVUS Biologicals (USA).

### Immunohistochemistry Examination

Immunohistochemistry was used to detect the expression of human MHC class I+HLA A+HLA B and alpha-smooth muscle actin (α-SMA) in the rabbit cranial defects to show local HAMSC survival and vascular integrity in the defect area. The sections were deparaffinized by xylene, hydrated with gradient ethanol, performed heat mediated antigen retrieval in citrate buffer, pH 6.0. Samples were then blocked by 5% sheep serum for 30 min and incubated separately with primary antibody human MHC class I+HLA A+HLA B (1:100, ab134189, Abcam, UK), α-SMA (1:200, ab7817, Abcam, UK) in a wet box at 4°C overnight. Consequently, samples were washed three times with PBS for 5 min per time and HRP-conjugated secondary antibody (MXB Biotechnologies, China) at room temperature for 20 minutes. Finally, samples were washed with PBS 3 times for 5 minutes per time, developed by DAB, microscopically observed, and photographed.

### Sequential Fluorescent Labeling

The polychrome sequential labeling of mineralizing tissues was performed to display new bone formation and mineralization. At 3, 6, and 9 weeks after the operation, the rabbits in the 12-week group were subjected to intraperitoneal injection of fluorochromes under anesthesia as follows: 20 mg/kg calcein (CA, Sigma, USA) 13, 30 mg/kg alizarin red S (AL, Sigma, USA) 13 and 90 mg/kg xylenol orange (XY, Sigma, USA) 15. The sections were examined under the confocal laser scanning microscope (ZEISS, Germany). Excitation/emission wavelengths of the fluorescent dyes were 488/517 nm (CA), 543/617 nm (AL) and 440/570 nm (XY).

### Statistical Analysis

All the data were expressed as means ± standard deviation (SD) and were analyzed using one-way ANOVA statistical analysis to evaluate the significance of the experimental data. *: p < 0.05, **: p < 0.01.

## Results

### Proteome Profile of HAMSCs and HBMSCs

**Figure 1:**
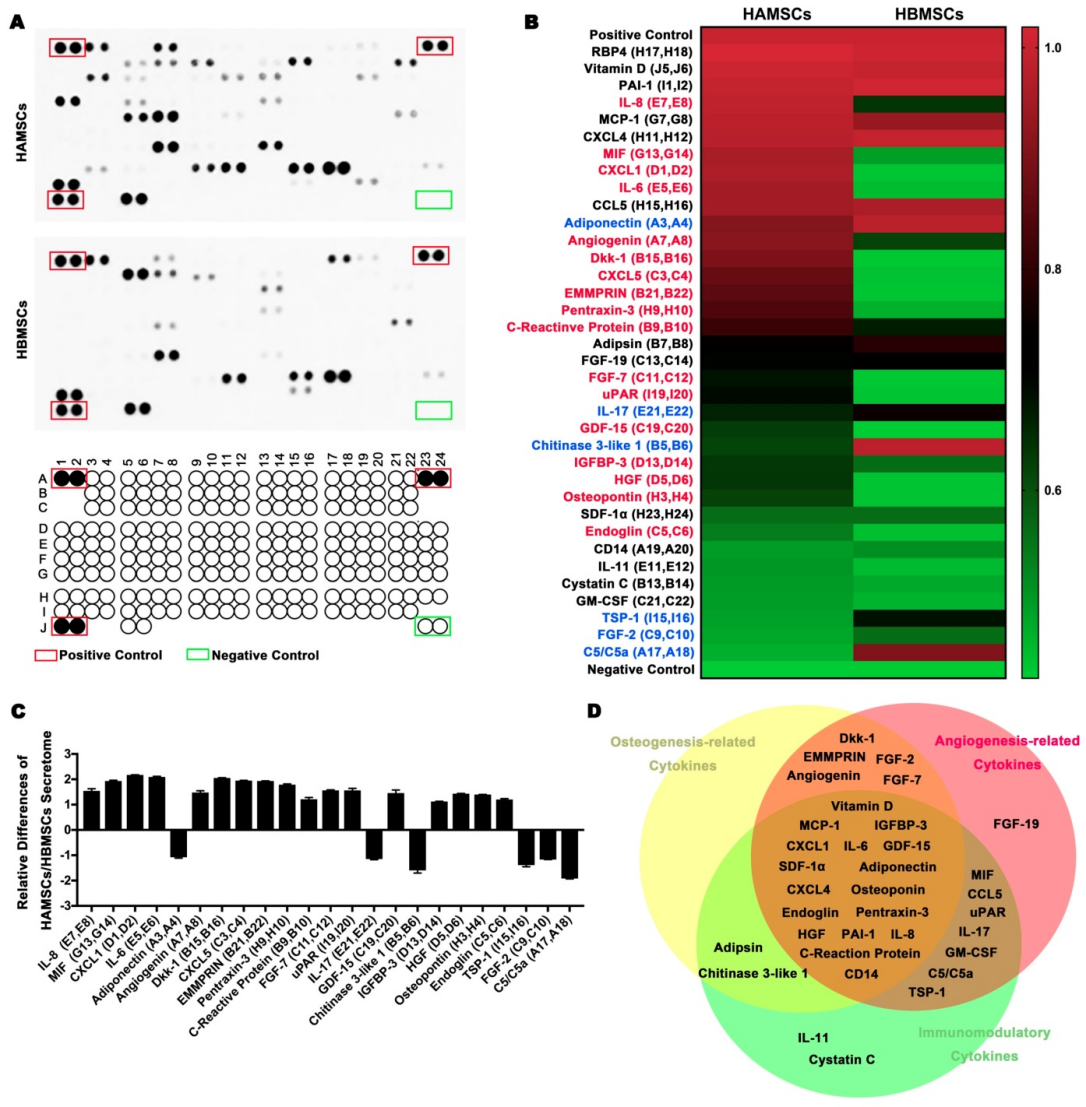
** Analysis of cytokines from HAMSCs and HBMSCs.** (A) Arrays of HAMSCs and HBMSCs, Schematic diagram of Human XL Cytokine Array Coordinates. (B, C) Different proteome profiles of HAMSCs and HBMSCs; expression of 17 cytokines of HAMSCs were higher than those of HBMSCs (labeled in red), while six cytokines were lower (labeled in blue). (D) Classification of cytokines secreted by HAMSCs.

To investigate the paracrine function of HAMSCs on regulating bone defect microenvironment, we collected HAMSC-conditional media to examine its proteome profile and compare it with the HBMSC's profile to determine their differences between them. Figure 1A showed multiple cytokines, chemokines, growth factors and other soluble proteins in HAMSC- and HBMSC- conditional media, which were listed in Figure 1B. We compared corresponding signals on the two arrays, and found significant differences in the expressions of 23 bioactive factors (Figure 1B and 1C). Among these, the expressions of 17 cytokines of HAMSCs were higher than those of HBMSCs (labeled in red, Figure 1B), while six cytokines were lower (labeled in blue, Figure 1B). We classified all bioactive factors into osteogenesis-related, angiogenesis-related, and immunomodulatory cytokines in Figure 1D.

### Osteoinduction and Chemotaxis of HAMSCs for HBMSCs

**Figure 2:**
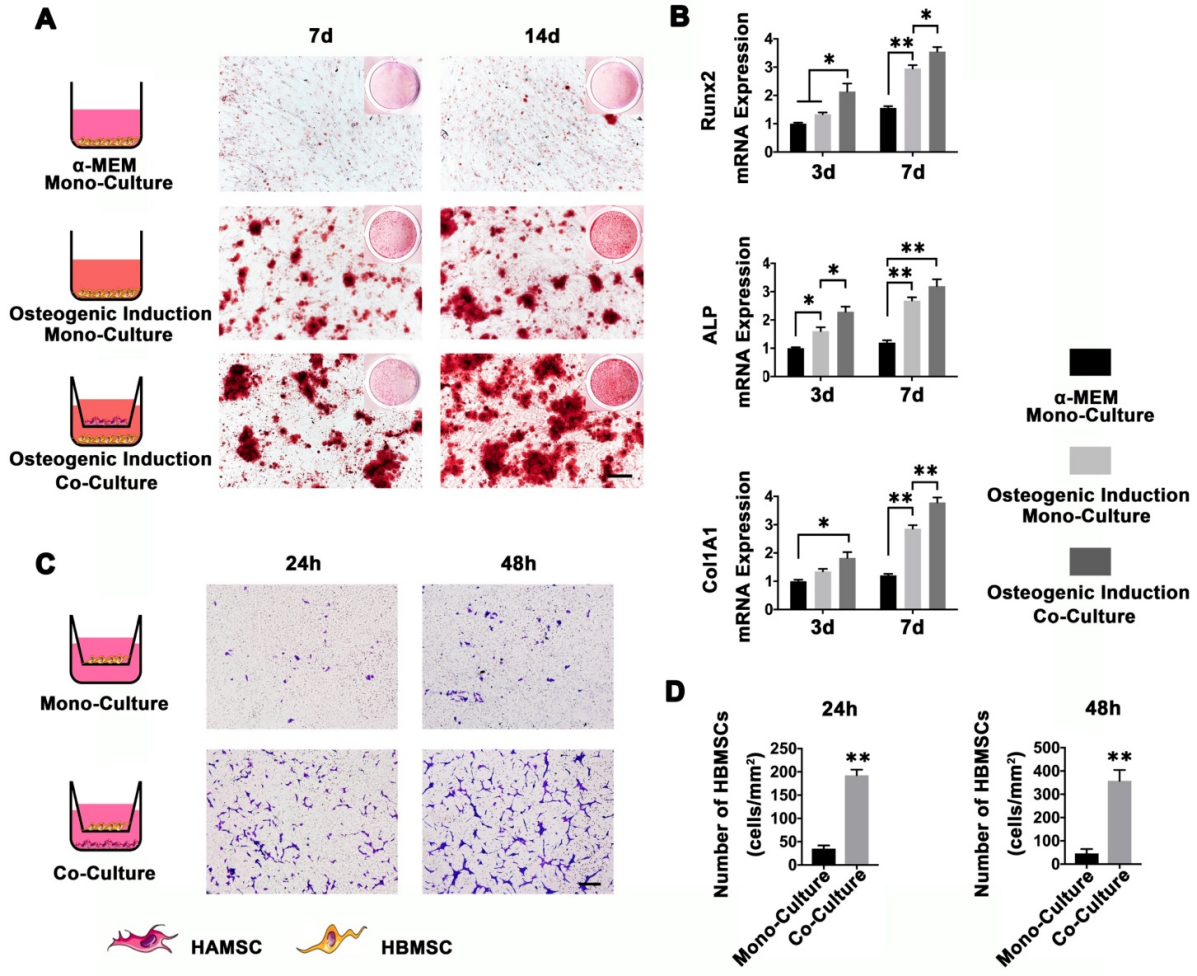
** HAMSCs Osteoinduction and Chemotaxis for HBMSCs.** (A) Alizarin red S staining of HBMSCs in different culture conditions (Scale bar: 200μm). (B) Relative Runx2, ALP and Col1A1 mRNA expressions of HBMSCs in different culture conditions. (C, D) Migration assay of HBMSCs in mono-culture and co-culture conditions. (Scale bar :200μm; ⁎: p<0.05; ⁎⁎: p<0.01).

To determine whether HAMSCs could recruit HBMSCs to the defect areas and enhance host BMSCs osteo-differentiation, we used a transwell system to co-culture HAMSCs and HBMSCs. As shown in Figure 2A, HBMSCs were stained by Alizarin red S at 7d and 14d after osteogenic induction. The results showed that HBMSCs could form obvious mineralized nodules. When co-cultured with HAMSCs, the mineralized nodules formed by HBMSCs become even more obvious compared to the mono-culture. We then detected osteogenic markers of HBMSCs by PCR. The results showed the relative Runx2, ALP and Col1A1 mRNA expressions of HBMSCs were significantly upregulated by HAMSCs after 3d and 7d co-culture (Figure 2B). On the other hand, HAMSCs could induce HBMSCs to migrate across the transwell membrane. After 24h co-culture, the number of HBMSCs in each field was markedly higher than that in mono-culture. The same trend could be observed after 48 co-culture (Figure 2C and 2D). These data demonstrated HAMSCs could recruit HBMSCs to promote osteogenesis.

### Angiogenesis and Chemotaxis of HAMSCs for HUVECs

**Figure 3:**
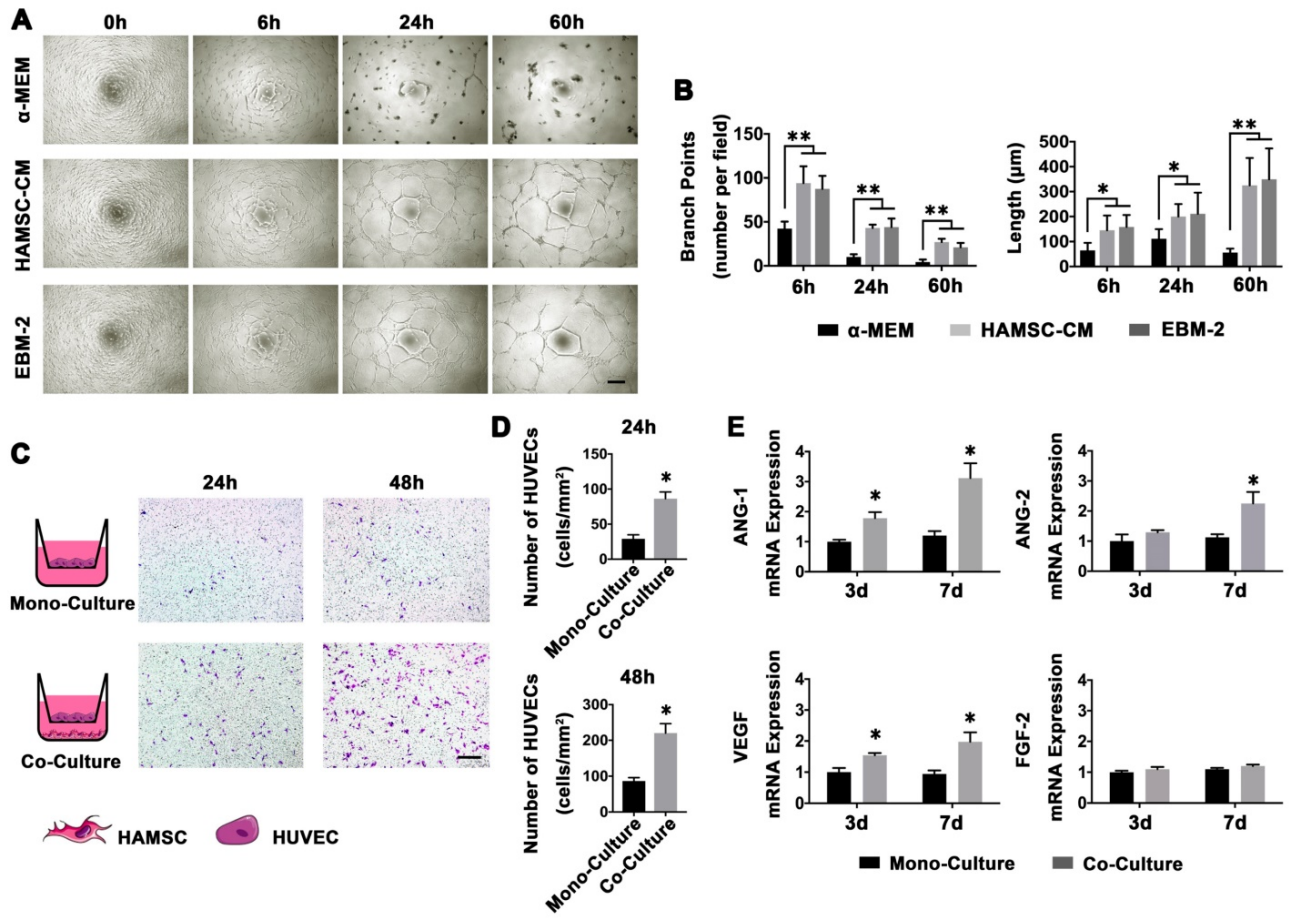
** Angiogenesis and Chemotaxis of HAMSCs for HUVECs.** (A) Capillary-like tube formation assay of HUVECs in different culture conditions (Scale bar: 200μm). (B) Branch points and length of HUVEC-sprouting. (C, D) Migration assay of HUVECs in mono-culture and co-culture conditions (Scale bar: 200μm). (E) Relative ANG-1, ANG-2, VEGF and FGF-2 mRNA expressions of HUVECs in mono-culture and co-culture conditions. (⁎: p<0.05; ⁎⁎: p<0.01).

Blood vessels play a key role in bone regeneration. Thus, we investigated whether HAMSCs could recruit and promote host HUVECs to form a capillary network. The endothelial basal medium-2 (EBM-2) contains a variety of cytokines (including VEGF, FGF2, EGF, Vitamin C and so on) which are needed for in vitro culture of HUVECs, but a-MEM contains no cytokines. HUVECs can achieve capillary-like tube formation assay in EBM-2, but not in α-MEM. Thus, α-MEM serves as a negative and EBM-2 as positive control. We cultured HAMSCs without serum for 48h and collected the HAMSC-CM to perform HUVECs capillary-like tube formation assay. HUVECs cultured in α-MEM were unable to form capillary-like tubes on Matrigel. However, when they cultured in the other two conditions, they formed stable capillary-like tubes on Matrigel at 6h; the area of tubes then gradually increased and the sprouting gradually became longer (Figure 3A). There were no statistical differences in branch points of HUVEC-sprouting and length of HUVEC-sprouting between the HAMSC-CM group and the EBM-2 group (Figure 3B).

The HAMSCs also could induce HUVECs to migrate across the transwell membrane. After 24h co-culture, the number of HUVECs in each field was markedly higher than that in mono-culture. The same trend could be observed after 48 co-culture (Figure 3C and 3D).

Next, we tested the angiogenesis-related mRNA expression of HUVECs under co-culture with HAMSCs and mono-culture conditions. The PCR results showed that the relative VEGF and angiopoietin-1 (ANG-1) mRNA expressions of HUVECs were significantly upregulated by HAMSCs after 3d and 7d co-culture, while the ANG-2 expression was upregulated after 7d co-culture (Figure 3E). These data demonstrated HAMSCs could recruit HUVECs to promote angiogenesis.

### Role of HAMSCs on Macrophage Polarization

**Figure 4:**
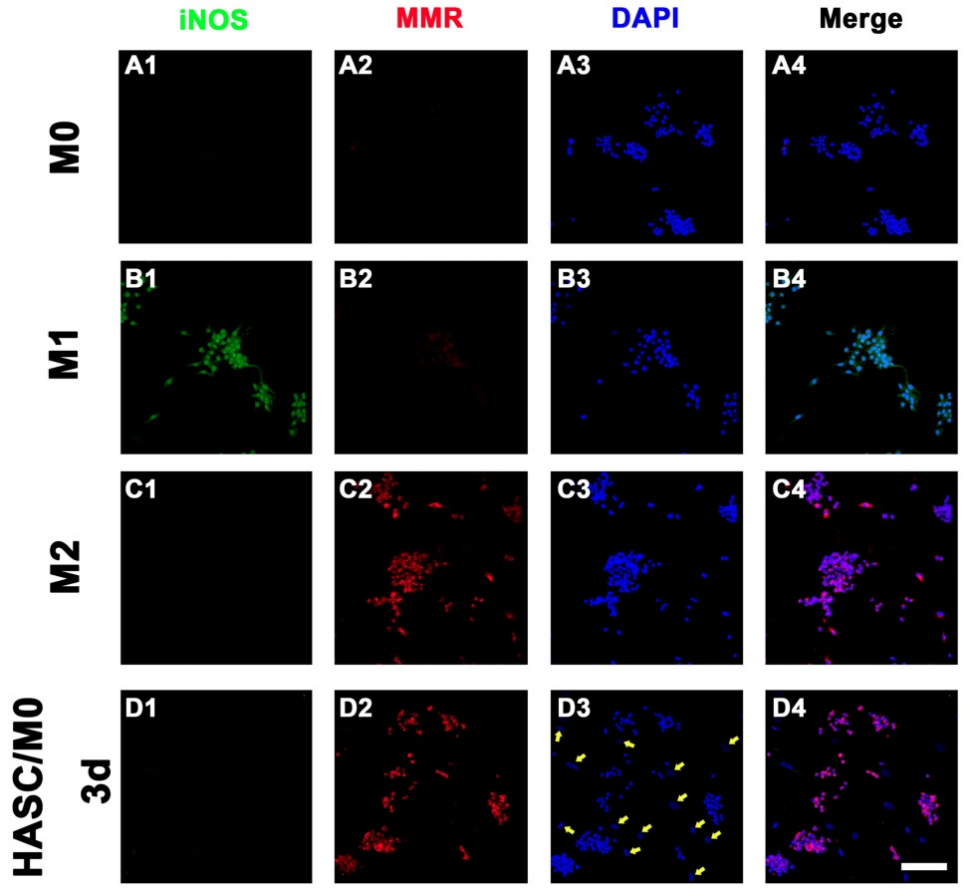
** The role of HAMSCs on Macrophage Polarization.** The iNOS and MMR expression of M0 group (A), M1 group (B), M2 group (C), and HAMSC/M0 group (D) (Scale bar: 50μm).

Macrophages have a crucial role in vascularized bone regeneration. The functional plasticity of macrophages has been conceptualized as macrophage polarization. To detect whether HAMSCs could induce macrophage polarization, we directly co-cultured HAMSCs with M0 macrophages for 3d and discovered that the M0 macrophages were polarized to M2 macrophages. As shown in Figure 4D2, positive expression of macrophage mannose receptor (MMR, anti-inflammatory M2 markers) was detected in HAMSC/M0 co-culture group by immunofluorescence staining. In addition, HAMSCs nuclei were larger than those of M2 macrophages (the nuclei of HAMSCs are indicated by yellow arrows, Figure 4D2). Thus, we believed that HAMSCs could induce M2 polarization to inhibit inflammation caused by macrophages and indirectly promote vascularized tissue regeneration.

### Survival of Transplanted HAMSCs in Early Stage

**Figure 5:**
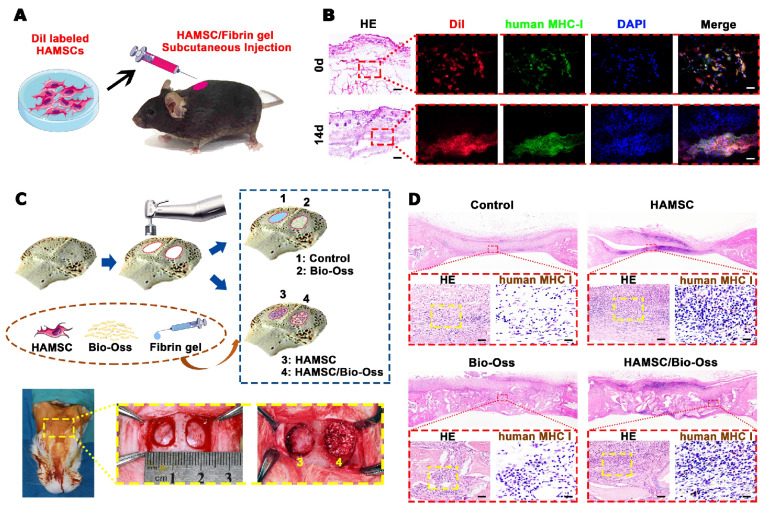
** Survival of Transplanted HAMSCs in Early Stage.** (A) Schematic illustration of HAMSC/ fibrin gel subcutaneous injection. (B) Histological and human MHC I immunofluorescent staining of subcutaneous tissue after HAMSC/ fibrin gel injection (Scale bar, HE: 400μm; Immunofluorescent staining: 100μm). (C) Schematic illustration and surgical images of rabbit cranial defects. (D) Histological and human MHC I immunohistochemical staining of rabbit cranial defects from four groups (Scale bar, HE: 100μm; Immunohistochemical staining: 50μm).

The survival of the transplanted HAMSCs was undoubtedly critical to the paracrine function in local trauma regions within the first two weeks usually defined as an acute inflammatory period. Firstly, Dil-labeled HAMSCs were mixed in fibrin gel and injected subcutaneously into C57BL/6 mice. After 14d, the fibrin gel was absorbed, but the Dil-labeled cells could still be seen under the skin with positive expression of human-specific marker (Figure 5B). Then, we introduced HAMSCs into cranial bone defects of rabbits. We surprisingly found positive expression of human MHC class I+HLA A+HLA B (human MHC I) in the 2-week samples from two groups containing HAMSCs (Figure 5D). These data proved that transplanted HAMSCs could survive during the early stage of tissue regeneration.

### Role of HAMSCs in an early period of cranial defect healing

**Figure 6:**
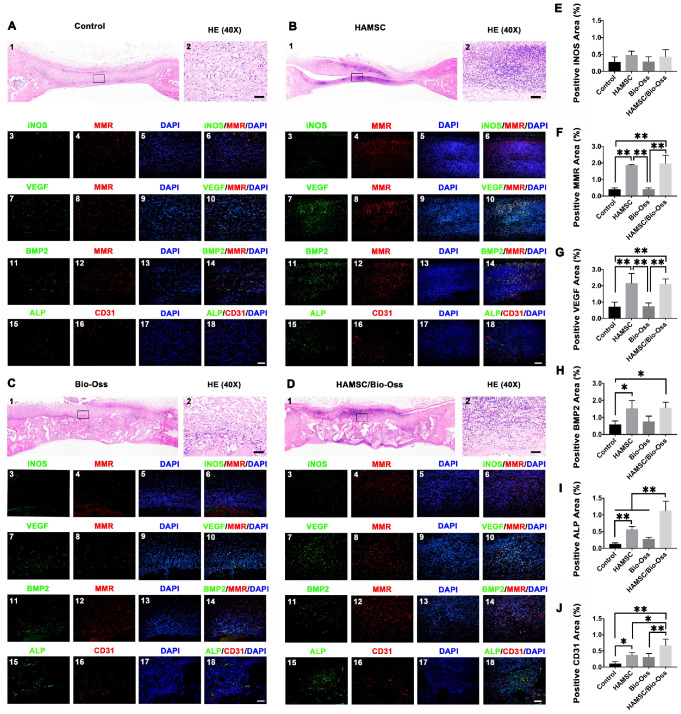
** Histological and immunofluorescent staining of 2-week groups.** (A-D) HE staining and immunofluorescent staining (iNOS, MMR, VEGF, BMP2, ALP and CD31) of the Control group, HAMSC group, Bio-Oss group and HAMSC/Bio-Oss group (Scale bar, HE: 400μm; Immunofluorescent staining: 100μm). (E) Positive iNOS area of four groups. (F) Positive MMR area of four groups. (G) Positive VEGF area of four groups. (H) Positive BMP2 area of four groups. (I) Positive ALP area of four groups. (J) Positive CD31 area of four groups. (⁎: p<0.05; ⁎⁎: p<0.01).

To investigate whether HAMSCs could be beneficial to improve the microenvironment of bone defects at the early healing stage, we evaluated the infiltration of macrophages in the four groups (Control group, HAMSC group, Bio-Oss group, HAMSC/Bio-Oss group) by immunofluorescence staining of the samples from the 2-week group. In Figure 6, HE staining showed the general situation in the four groups respectively. Dense cell infiltration was observed in the HAMSC group and HAMSC/ Bio-oss group (Figure 6A1, B1, C1, D1). We intercepted and amplified the local areas with dense cell infiltration, and performed HE staining (Figure 6A2, B2, C2, D2) and macrophage markers staining to confirm that the most cells were the M2 macrophages (MMR positive), as shown in Figure 6A4, B4, C4, D4 and 6F. Following these results, we co-stained MMR (M2 macrophage marker) with VEGF and BMP2 to determine whether M2 macrophage could enhance vascularized bone regeneration in our samples. As expected, we found that M2 macrophages could produce VEGF and BMP2 (Figure 6B11, B11, D11 and D11). There were more VEGF and BMP2 positive expressions in two groups that contained HAMSCs (Figure 6G and 6H). Besides, both ALP positive cells and CD31 positive vascular structures were more in these groups (Figure 6B18 and 6D18). The transplanted HAMSCs could regulate M2 infiltration in bone healing microenvironment to promote the osteogenesis related precursor cells migration. The positive ALP and CD31areas of two groups that contained HAMSCs were significantly higher than those of the control group. The regenerative effect of HAMSC was further confirmed by statistical analysis (Figure 6I-6J).

### Osteogenic effect of 4-week and 12-week groups

#### Radiographic Analysis of 4-week and 12-week groups

**Figure 7:**
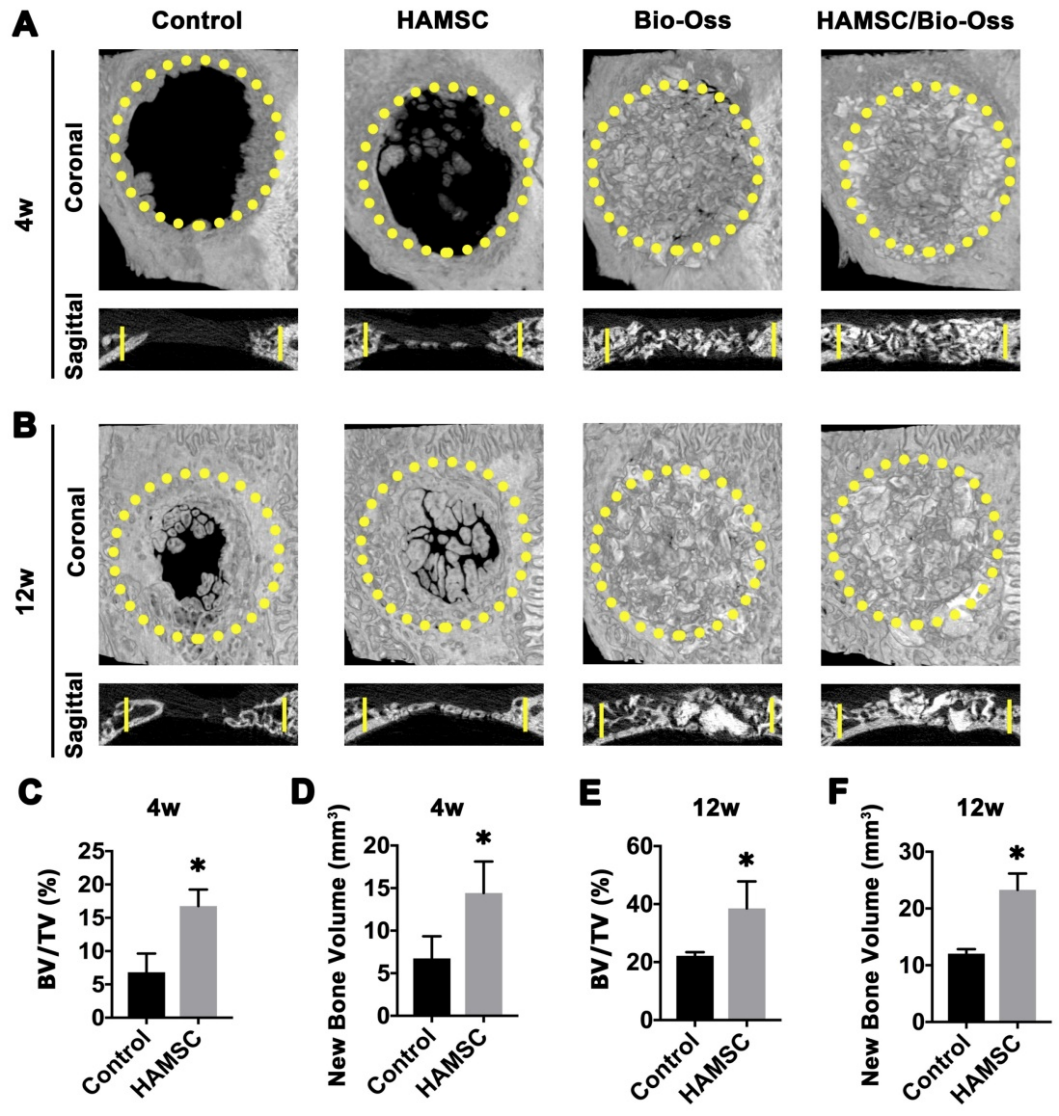
** Radiographic Analysis of 4-week and 12-week groups.** (A) X-Ray images of the Control group, HAMSC group, Bio-Oss group and HAMSC/Bio-Oss group at the 4-week timepoint; (B) X-Ray images of the four groups at the 4-week timepoint; (C, D) BV/TV and new bone volume of the Control group and HAMSC group at the 4-week timepoint; (E, F) BV/TV and new bone volume of the Control group and HAMSC group at the 12-week timepoint. (⁎: p<0.05; ⁎⁎: p<0.01).

To observe new bone formation within the defects, X-Ray images were taken at 4, 12 weeks after the operation. Representative images of four groups are shown in Figure 7A and 7B. Since Bio-Oss is a kind of radiopaque graft materials, we detected and analyzed new bone formation in two contained Bio-Oss groups by subsequent histological staining. At four weeks, edges of the defect areas displayed irregular grainy in all groups. More scattered radiopaque areas were detected in the HAMSC group. In 12-week groups, the cranial defects in the HAMSC group were covered with newly formed X-Ray obstructive tissue, which was not observed in Control group. Bone volume (BV) and Bone volume/Tissue Volume (BV/TV) of HAMSC group (14.42
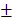
3.712 mm^3^, 16.75
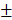
2.483%) were significantly higher than those of control group (6.747
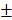
2.603mm^3^, 6.833
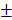
2.802%) in 4-week timepoint. In 12-week timepoint, BV and BV/TV of HAMSC group (23.29
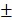
2.869 mm^3^, 38.51
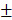
9.302%) were also significantly higher than those of control group (12.04
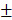
0.809mm^3^, 22.16
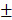
1.271%) (Figure 7C-7F).

#### Histological Analysis of 4-week and 12-week groups

**Figure 8:**
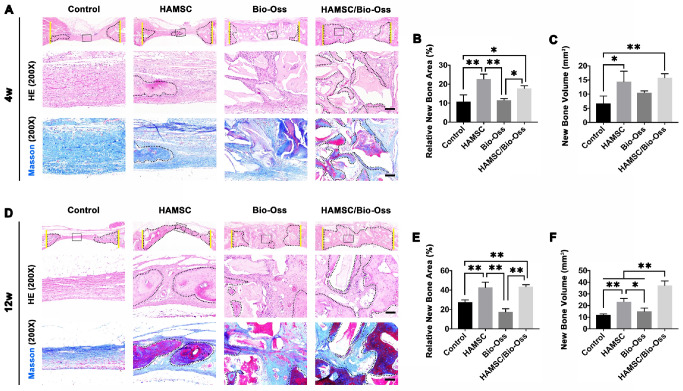
** Histological Analysis of 4-week and 12-week groups.** (A) HE staining and Masson's trichrome staining of the Control group, HAMSC group, Bio-Oss group and HAMSC/Bio-Oss group at the 4-week timepoint (Scale bar, HE: 100μm; Masson's trichrome staining: 100μm). (B, C) Relative new bone area and new bone volume of the four groups at the 4-week timepoint. (D) HE staining and Masson's trichrome staining of the four groups at the 12-week timepoint (Scale bar, HE: 100μm; Masson's trichrome staining: 100μm). (E, F) Relative new bone area and new bone volume of the four groups at the 12-week timepoint. (⁎: p<0.05; ⁎⁎: p<0.01).

After analyzing the phenotype of the samples in 2w-groups, we further observed the osteogenic effect in 4w-groups and 12w-groups. As shown in Figure 8A and 8D, the dotted line outlined the area where the new bone has formed. We found new bone tissue had formed from the edge of defects to the center. In the 4-week timepoint, Bio-Oss particles seemed to impede bone formation in the defects, but the Bio-Oss poor osteoinduction was improved by HAMSCs. The relative new bone area and new bone volume of HAMSC/Bio-Oss group (17.73
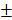
1.466%, 15.75
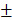
1.458mm^3^) were significantly higher than those of Bio-Oss group (11.59
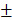
0.76%, 10.51
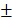
0.648mm^3^) (Figure 8B and 8C). As time went by, the new bone of the two groups containing transplanted HAMSCs was observed in the whole defect area at 12 weeks after operation, the relative new bone area and new bone volume of HAMSC group and HAMSC/Bio-Oss group were 42.83
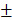
5.36%, 43.4
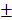
2.203% and 23.29
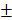
2.869mm^3^, 37.18
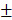
3.96mm^3^, respectively, and were significantly higher than those of Control group and Bio-Oss group (Figure 8E and 8F). In addition, Masson's trichrome staining showed HAMSCs not only accelerated the new bone formation, but promoted new bone maturation (the mature bone was stained as “wine” red in Masson's trichrome staining). From the radiographic and histological analysis, we considered transplanted HAMSCs could enhance osteogenesis in bone defects.

### Neovascularization of 2-week, 4-week and 12-week groups

**Figure 9:**
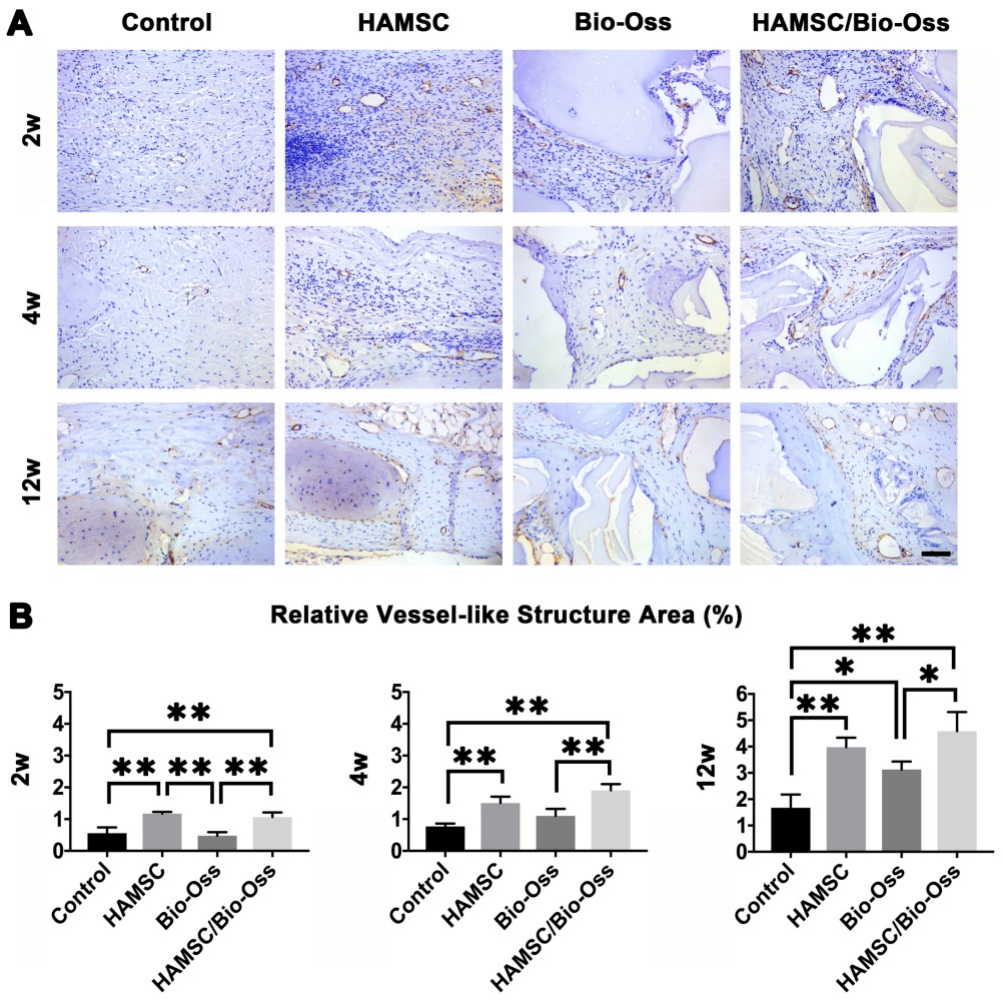
** The neovascularization of 2-week, 4-week and 12-week groups.** (A) α-SMA immunohistochemical staining of the Control group, HAMSC group, Bio-Oss group and HAMSC/Bio-Oss group at 2-week, 4-week and 12-week timepoints (Scale bar: 100 μm). (B) Relative vessel-like structure area of the four groups at three timepoints. (⁎: p<0.05; ⁎⁎: p<0.01).

Local vascularization in the bone defect area is considered to be quite important for bone regeneration. In this study, we continuously explored vascular penetration and integrity of blood vessels during bone defect healing. The α-SMA, which can be found in the vessel wall, is located primarily in the microfilament bundles of vascular smooth muscle cells (VSMCs) where it exerts contractile functions. We found that the quantities of vessel-like structures (α-SMA positive staining structures) in the HAMSC group and HAMSC/Bio-Oss group at 2w were higher than those in Control group and Bio-Oss group. At 4w and 12w, vessel-like structures could be found to accompany with new bone in HAMSC group and HAMSC/Bio-Oss group. The quantities of the structures were also more than those of two other groups. In 12-week groups, we observed a large number of blood vessels with complete α-SMA-positive vascular walls in groups containing transplanted HAMSCs (Figure 9). Blood vessels with a complete tubular wall suggested that vessels have a normal vascular function in providing nutritional support for the defects repairing.

### Bone Metabolism and Mineralization at 3, 6 and 9 weeks

**Figure 10:**
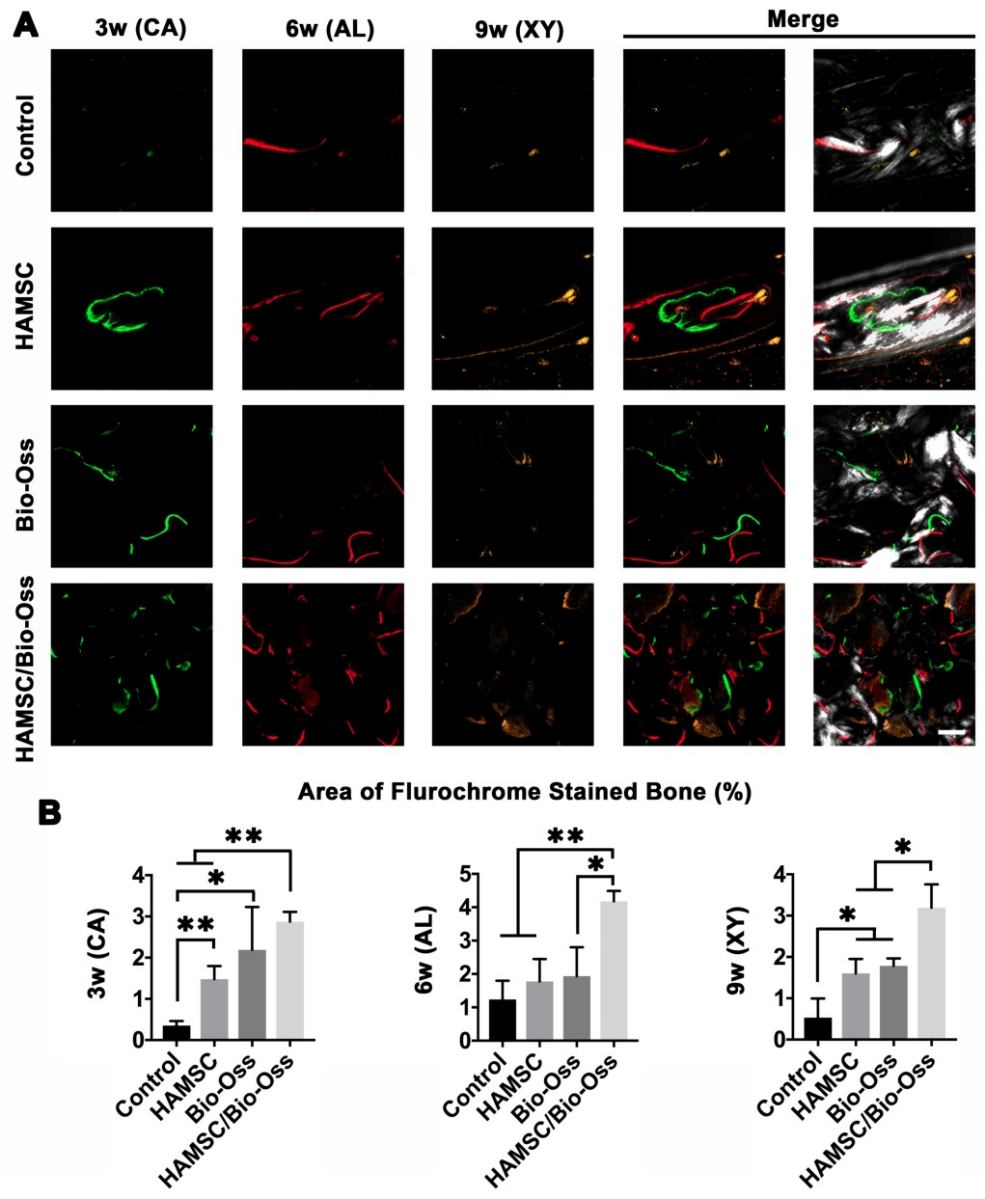
** Polychrome sequential fluorescent labeling.** (A) Calcein (CA), Alizarin red (AL), and Xylenol (XY) merged images of the Control group, HAMSC group, Bio-Oss group and HAMSC/Bio-Oss group at different timepoints (Scale bar: 200 μm). (B) Relative area of fluorochrome stained bone of the four groups at different timepoints. (⁎: p<0.05; ⁎⁎: p<0.01).

Dynamic bone histologic analyses were evaluated by fluorescent labeling measurements (Figure 10A). The fluorescent labeling showed that the percentage of calcein (CA), alizarin red (AL) and xylenol orange (XY) positivity in the Bio-Oss/HAMSC group (2.875 ± 0.237%, 4.178 ± 0.311% and 3.192 ± 0.565%) was significantly higher than that of the other three groups (Figure 10B). These results confirmed that HAMSCs improved bone formation and accelerated consistent bone mineralization in bone defects.

## Discussion

So far, many strategies for reconstruction of complex skeletal defects have been developed, such as autologous bone transplantation and the application of bioactive factors and/or bio-materials), Yet, the major shortcomings of those methods are limited sources of autologous bone, increased patient's pain, uncertain efficacy of biomaterials, and high treatment cost 16-18. Stem cell-based therapy is an alternative approach that can be used to solve these problems.

The paracrine function of MSCs can provide active factors needed for tissue regeneration 19. It has been accepted by increasing researchers that the healing process and the inherent repair capacity of skeletal tissues are related to soluble signals, which regulate the recruitment of endogenous cells to the injury sites 19, 20. At the beginning of this study, we analyzed paracrine functions of HBMSCs and HAMSCs and found that the secretion profiles and levels of soluble factors in the two cells were different. The expressions of 17 cytokines of HAMSCs were higher than those of HBMSCs. Among those cytokines, IL-6 21, IL-8 22, CRP and Pentraxin-3 23 have been reported to have a suppressive effect on innate immune cells (macrophages, neutrophils and NK cells). MIF 24 and osteopontin 25 can regulate and create an immunosuppressive microenvironment by inhibiting neutrophil infiltration and macrophage accumulation, while GDF-15 contributes to escape from macrophage immune-surveillance 26. From the aspect of recipient adaptive immunity, IL-6, IL-8, CXCL1, HGF, and IGFBP3 can be released by MSCs to inhibit T-cell proliferation and infiltration 27, 28. CXCL5 has been certified to ameliorate graft-versus-host disease (GVHD) via suppression of Th 1 and Th 17 responses via *in vitro* and *in vivo* data 29. The upregulation of EMMPRIN could result in dysfunction of immune cells including T cells, and the consequent immunological hyporesponsiveness 30. Higher expression of those cytokines in HAMSCs suggested that HAMSCs had superior immune regulation functions, which were beneficial for the cells to escape host immune monitoring and suppress the local immune response. As shown in Figure 5, HAMSCs were able to survive for at least 2 weeks after transplantation. These results might provide researchers with convincing evidence on the clinical efficacy of HAMSC-transplantation.

During a bone healing process, inflammation, angiogenesis, and new bone regeneration are intimately linked. Inflammatory monocytes and resident tissue macrophages are not only key regulators in bone repair 31, but are crucial factors in inducing endogenous regeneration. Macrophages can be generated from monocytes and undergo classical (M1) or alternative (M2) activation 32. It is believed that the polarization of macrophage phenotype towards the anti-inflammatory M2, rather than the inflammatory M1 phenotype, promotes osteogenesis 33, 34. Yet, the exact effect of HAMSCs on the polarization of macrophage is not well understood. In this study, we examine the process by co-culturing macrophage cells with HAMSCs cells. After three days of co-culture, we found the polarization of RAW264.7 (macrophage cell line) towards M2 *in vitro* (Figure 4). The infiltration of M2 macrophages was observed in the samples of the two groups with HAMSCs two weeks after operation (Figure 6). Immunofluorescent staining data indicated that M2 macrophages expressed VEGF and BMP2. Previous studies have reported M2 macrophages could express VEGF and BMP2 to induce endogenous bone regeneration 35, 36. Our data showed the mechanisms of HAMSCs promoting osteogenesis should be involved in polarizing M2 macrophages in bone defects to stimulate endogenous regeneration. Besides the regulatory functions on host macrophages, HAMSCs secreted cytokines also have directly significant pro-angiogenic and pro-osteogenic functions, such as IL-6, IL-8, angiogenin, CXCL1, CXCL5, HGF, FGF-7, vitamin D and osteopontin. We co-cultured HAMSCs with HUVECs and expectedly found that HAMSCs promoted HUVECs migration and up-regulated the pro-angiogenic genes of HUVECs (Figure 3). When we co-cultured HAMSCs with HBMSCs, HBMSCs migrated faster and formed more mineralized nodules. Their expression of some osteogenic genes was up-regulated (Figure 2). As shown by the corresponding *in vivo* results in Figure 6, there were more positive areas of ALP and CD31 in groups with HAMSCs compared to the Control group and Bio-Oss group. These data supported that HAMSCs could recruit the related cells to enhance vascularized bone regeneration in the early period. The following osteogenic effects of HAMSCs were shown in Figure 7, Figure 8, Figure 9 and Figure 10.

Bio-Oss is one of the most common bone-grafting materials used in clinical practices, including periodontal surgery 37, alveolar surgery and oral implantation 38. In this study, Bio-Oss was selected to serve as a positive control. Some previous reports have reported that Bio-Oss particles had poor osteoinductive properties; and should only be used as a scaffold to occupy the space needed for regeneration 39, 40. In the present study, we found poor bone formation in Bio-Oss groups by comparing four groups in 4-week and 12-week time points by histological staining, which was consistent with the above-mentioned studies 40, 41. Next, we investigated whether HAMSCs could improve the osteogenic efficacy of Bio-Oss. Briefly, we found that bone volume and BV/TV of HAMSC/Bio-Oss group in 4 weeks and 12 weeks were markedly higher than those of Bio-Oss groups and the other two groups (Figure 8). In addition, the bone metabolic activity reflected by three indicate markers (CA, AL and XY), and the vascular maturity reflected by α-SMA also showed that the application of HAMSCs could significantly promote the vascularized bone regeneration from multiple perspectives. As shown in our results, the combined application of HAMSCs and Bio-Oss particles in the bone defect areas might be a good option to make up for the poor osteogenic effect by using Bio-Oss alone in current clinical practice.

## Conclusions

HAMSCs can secrete a variety of osteogenic, angiogenic cytokines, thus promoting neovascularization and osteogenesis. In addition, HAMSCs can release immunomodulatory factors to polarize M0 macrophages into M2 anti-inflammatory/wound healing macrophages, thus improving local microenvironment of bone defects. In summary, HAMSCs may be used as an alternative approach to bone tissue regeneration in clinic.
