# Supplementary material for: Human amniotic mesenchymal stromal cells promote bone regeneration via activating endogenous regeneration
Source: Theranostics. 2020 May 15;10(14):6216–30. doi: 10.7150/thno.45249 (PMC7255030; doi:10.7150/thno.45249)
Supplement: Supplementary file 1 — Supplementary figures and tables. [file thnov10p6216s1.pdf]

Supplementary Material

Methods

Cell culture

Isolation and culture of HAMSCs

The HAMSCs were isolated following a previous protocol[1]. Human term placentas were harvested from normal pregnancies (range 38 to 41 weeks) after spontaneous delivery or cesarean section with informed consent. Approval of the Ethical Committee of Nanjing Medical University was granted (No. PJ2014-79-001). Briefly, the decidua parietalis and blood clots were carefully removed. The amnion was cut into small pieces (1cm×1cm) and washed in phosphate-buffered saline containing 100 U/ml penicillin and 100 μg/ml streptomycin (Beyotime, China). Amnion fragments were then incubated for 10 min at 37℃ in PBS containing 2.4 U/ml dispase (Roche, Germany). The incubated fragments were transferred to α-MEM (GIBCO, USA) supplemented with 10% heat-inactivated fetal bovine serum (FBS; GIBCO, USA) for resting 5-10min at room temperature. After the resting period, the fragments were digested with 0.75 mg/ml collagenase (Roche) and 20 μg/ml DNAse (Roche) for approximately 3 h at 37℃. After collagenase and DNAse digesting, fragments were removed and the cells were passed through a 100 μm cell strainer (B&D Falcon, USA) and collected by centrifugation at 200g for 10min. The cells were cultured in the α-MEM complete media and used before passage 6.

MSC-specific surface markers of HAMSCs

The MSC-specific surface markers of HAMSCs were tested by flow cytometry and their multi-differentiation capacity (Figure S1). The primary antibodies for flow cytometry were purchased from Miltenyi Biotec (Germany).

Multi-differentiation Potential of HAMSCs

The HAMSCs were induced to differentiate into adipocytes, osteoblasts and chondroblasts *in vitro*.

To induce osteogenic differentiation, HAMSCs were cultured in DMEM (Gibco, USA) supplemented with 10% FBS, 10 mM β-glycerophosphate, 0.2 mM ascorbic acid, and 10^{-8} M dexamethasone (Sigma, USA), and cultured for 21 days, replacing the medium every 3 days. To demonstrate osteogenic differentiation, the cultures were fixed in 4% PFA and stained by Alizarin Red S solution.

To induce adipocyte differentiation, HAMSCs were cultured in DMEM supplemented with 10% FBS, 0.5 mM IBMX (Sigma, USA), 200 μM indomethacin (Sigma, USA), 10^{-6}M dexamethasone and 10 μg/ml insulin (Sigma, USA) in chamber slides (NUNC, USA). The cells were cultured, replacing the medium every 2-3 days. After 21 days of culture, the cells containing neutral lipids in fat vacuoles were fixed in 4% PFA and stained by fresh Oil red-O solution (Sigma, USA).

To induce chondrogenic differentiation, aliquots of HAMSCs were pelleted in polypropylene conical tubes in 0.5 ml of DMEM containing 6.25 μg/ml insulin, 6.25 μg/ml transferrin, 6.25 μg/ml selenous acid, 5.33 μg/ml linolenic acid, 1.25 mg/ml BSA, 0.35 mM proline, 1 mM sodium pyruvate, 10^{-7}M dexamethasone, 0.1 mM L-ascorbic acid-2-phosphate (all Sigma, USA), supplemented with 10 ng/ml TGF-β 3

(R&D Systems, USA). This medium was replaced every three days for 28 days. Pellets were fixed in 4% PFA and stained by Toluidine Blue.

Isolation and culture of HUVECs

Human umbilical vein endothelial cells (HUVECs) were harvested from fresh umbilical cords following a previously established protocol[2]. After repeatedly washing the blood clots in the umbilical vein with PBS, 0.1% (w/v) collagenase type 1A solution was injected for digestion at 37℃ for 15min to release endothelial cells from the vessel walls. Then, the venous lumen was irrigated with EBM-2 (Lonza, Switzerland), and the digested HUVECs were collected by centrifugation at 1000rpm for 5min. HUVECs were cultured in EBM-2 and used before passage 5.

EC-specific markers of HUVECs

The EC-specific markers of HUVECs were tested by immunofluorescence (IF) staining and flow cytometry (Figure S2). The primary antibodies for flow cytometry were purchased from Miltenyi Biotec (Germany), while the antibodies (CD31, vWF) for IF staining were purchased from Abcam (UK).

Culture of HBMSCs

Human bone marrow-derived mesenchymal stem cells (BMSCs, passage 2) were purchased from Cyagen Biosciences Inc. (Guangzhou, China). The MSC-specific surface markers of these cells were tested by Cyagen Biosciences Inc. (Guangzhou, China). The company claims the HBMSCs were positive for CD105, CD29, and CD44 and negative for CD34, and CD45. The cells were cultured in the α-MEM complete media at 37℃.

Histological examination

All decalcified calvarial samples were placed in 70%, 80%, 90%, 95%, and 100% different concentrations of alcohol, and xylene was transparent, dipped in wax, embedded in wax blocks, and sliced 4 μm each slice, and then dewaxed for routine haematoxylin-eosin (H&E) staining and Masson's trichrome staining for collagen fibers. H&E and Masson's-trichrome-stained sections were observed with a light microscope for pathological changes.

The PCR primer sequences

Table S1

Runx2	F: TGGTTACTGTCATGGCGGGTA	R: TCTCAGATCGTTGAACCTTGCTA
ALP	F: ACTGGTACTCAGACAACGAGAT	R: ACGTCAATGTCCCTGATGTTATG
Col1A1	F: GTGCGATGACGTGATCTGTGA	R: CGGTGGTTTCTTGGTCGGT
Ang-1	F: AGCGCCGAAGTCCAGAAAAC	R: TACTCTCACGACAGTTGCCAT
Ang-2	F: AACTTTCGGAAGAGCATGGAC	R: CGAGTCATCGTATTCGAGCGG
VEGF	F: AGGGCAGAATCATCACGAAGT	R: AGGGTCTCGATTGGATGGCA
FGF-2	F: AGTGTGTGCTAACCGTTACCT	R: ACTGCCCAGTTCGTTTCAGTG

Results

Identification of hAMSCs

The hAMSCs were obtained by enzyme digestion. After 24 hours, the majority of the adherent cells stretched and became spindle or short-rod shaped. At passage three,

hAMSCs showed a fibroblast-like morphology (Figure S1A). Flow cytometry showed that hAMSCs expressed representative mesenchymal cell surface markers including CD29 (99.8%), CD44 (99.9%), CD105 (96.9%), CD90 (79.8%) and CD73b (99.6%). Human AMSCs were negative for CD34 (0.07%), CD45 (0.11%) and HLA-DR (0.04%) expression (Figure S1B-1L).

The hAMSCs at personal passage 3 could form many calcium deposits as shown by Alizarin Red S staining in osteogenic induction for 21days (Figure S1M). After 21 days of adipogenic induction, hAMSCs changed their morphological characteristics and accumulation of lipid droplets was detected by oil red O staining (Figure S1N). To test hAMSC chondrogenic differentiation, hAMSCs at personal passage3 were digested, centrifuged, and then pelleted and cultured in a chondrogenic induction solution. After 28 days the hAMSC-pellets formed cartilage-like spheres. The cartilage-spheres were subjected to cryo-sectioning and then stained with Toluidine Blue. The blue cartilage matrix was observed in the hAMSC-pellets (Figure S1O).

Identification of HUVECs

The HUVECs were obtained by enzyme digestion. After 24 hours, the majority of the adherent cells displayed “Ovoid” like shape (Figure S2A). Immunofluorescence staining showed that HUVECs expressed representative endothelial cell markers CD31 and vWF (Figure S2B and 2C). Flow cytometry showed that HUVECs were positive for CD34 (81.3%) and negative for CD45 (0.49%) (Figure S2G and S2H).

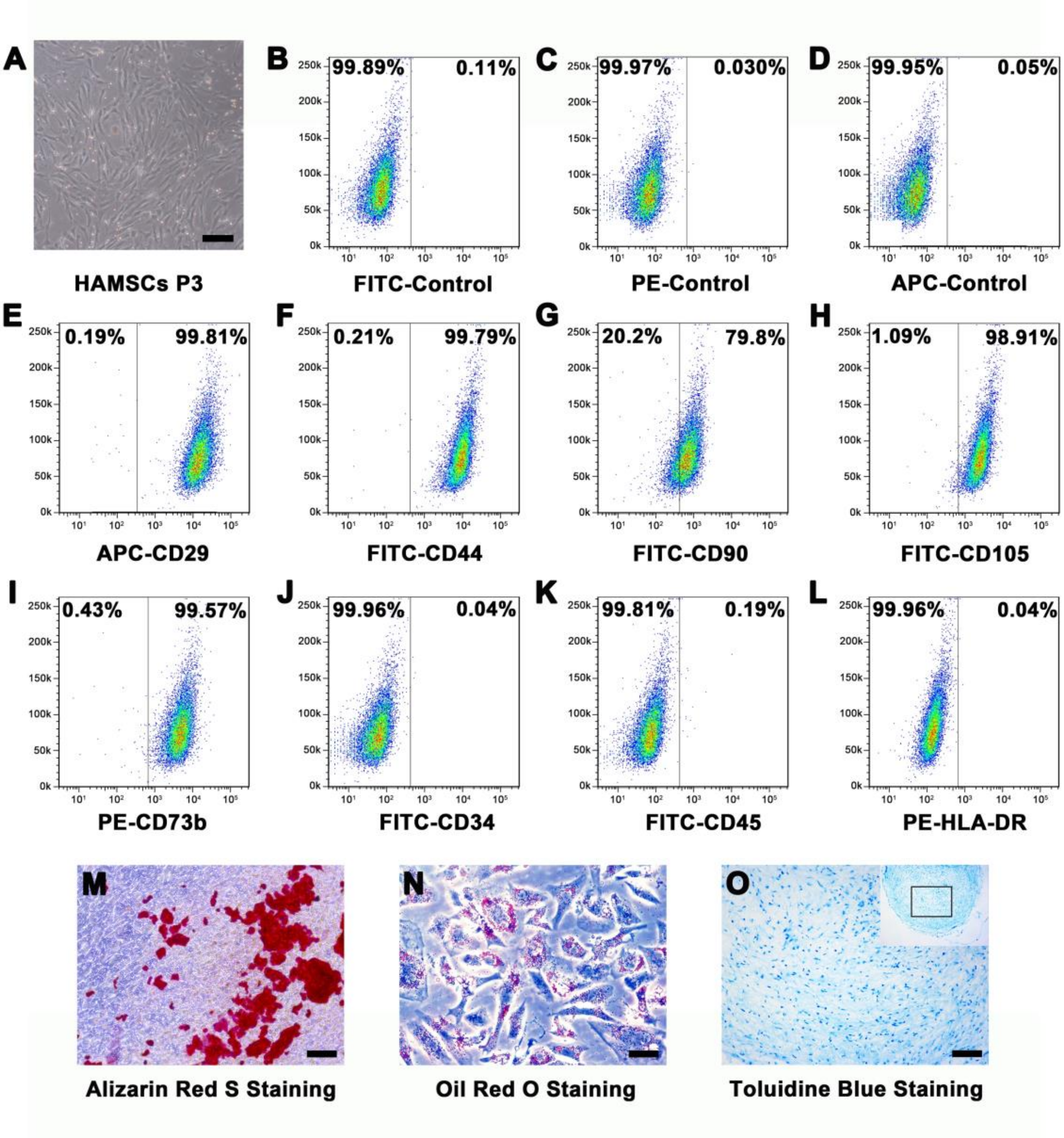

Supplementary Figure 1. Identification of HAMSCs. (A) Image of HAMSCs at passage 3 (Scale bar: 100μm). (B-D) Negative controls in each flow cytometric histogram; HAMSCs were positive for CD29 (E), CD44 (F), CD90 (G), CD105 (H), CD73b (I), and negative for CD34 (J), CD45 (K) and HLA-DR (L). (M) Alizarin Red S staining of HAMSCs at passage 3 (Scale bar: 50μm). (N) Oil Red O staining of HAMSCs at passage 3 (Scale bar: 50μm). (O) Toluidine Blue Staining of HAMSC-pellets (Scale bar: 100μm).

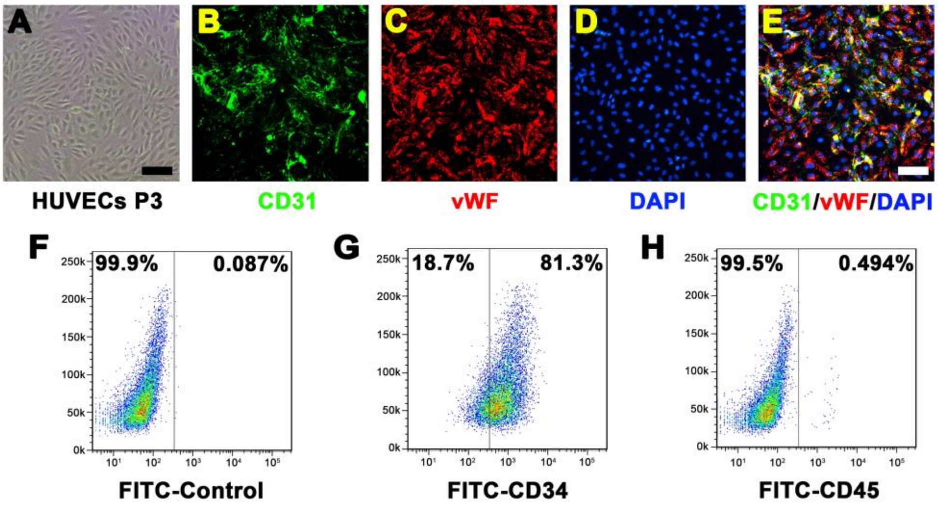

Supplementary Figure 2. Identification of HUVECs. (A) Image of HUVECs at passage 3 (Scale bar: 50μm). (B) Immunofluorescent staining of CD31 (Scale bar: 50μm). (C) Immunofluorescent staining of vWF (Scale bar: 50μm). (D) Immunofluorescent staining of DAPI (Scale bar: 50μm). (E) Merge image of CD31, vWF and DAPI (Scale bar: 50μm). (F) Negative control of FITC panel. (G) HUVECs were positive for CD34; and negative for CD45 (H).

References

1. Soncini M, Vertua E, Gibelli L, Zorzi F, Denegri M, Albertini A, et al. Isolation and characterization of mesenchymal cells from human fetal membranes. J Tissue Eng Regen Med. 2007; 1: 296-305.

2. Ghajar CM, Blevins KS, Hughes CC, George SC, Putnam AJ. Mesenchymal stem cells enhance angiogenesis in mechanically viable prevascularized tissues via early matrix metalloproteinase upregulation. Tissue engineering. 2006; 12: 2875-88.
